# Supplementary material for: Differential Proteomic Analysis of Low-Dose Chronic Paralytic Shellfish Poisoning
Source: Mar Drugs. 2024 Feb 26;22(3):108. doi: 10.3390/md22030108 (PMC10972186; doi:10.3390/md22030108)
Supplement: Supplementary file 1 [file marinedrugs-22-00108-s001.zip › marinedrugs-2761554-supplementary.pdf]

Table S1: List of all altered proteins in control and shellfish poisoning model groups.

NO.	Genename	Name	P-value	fold-change
1	Ezr	Ezrin	0.02	1.75
2	Scn1a	Sodium channel protein type 1 subunit alpha	0.05	1.27
3	Psap	Prosaposin	0.01	0.79
4	Park7	Parkinson disease protein 7	0.03	0.79
5	Gnai3	Guanine nucleotide-binding protein G(i) subunit alpha-3	0.02	1.26
6	St13	Hsc70-interacting protein	0.01	0.81
7	Myl6	Myosin light polypeptide 6	0.01	0.81
8	Clu	Clusterin	0.01	1.59
9	Tmod1	Tropomodulin-1	0.05	1.27
10	Hspe1	10 kDa heat shock protein, mitochondrial	0.02	0.83
11	Thnsl1	Threonine synthase-like 1	0.04	1.28
12	Cmpk2	UMP-CMP kinase 2, mitochondrial	0.04	1.35
13	Grik2	Glutamate receptor ionotropic, kainate 2	0.05	0.80
14	Slc38a3	Sodium-coupled neutral amino acid transporter 3	0.03	1.36
15	Fhip1b	FHF complex subunit HOOK interacting protein 1B	0.03	1.29
16	Nagk	N-acetyl-D-glucosamine kinase	0.04	1.27
17	Fmr1	Synaptic functional regulator FMR1	0.03	1.36
18	Gucy1a1	Guanylate cyclase soluble subunit alpha-1	0.04	1.21
19	Slc23a2	Solute carrier family 23 member 2	0.04	1.38
20	Nutf2	Nuclear transport factor 2	0.05	0.74
21	Pycr3	Pyrroline-5-carboxylate reductase 3	0.00	0.73
22	C5	Complement C5	0.03	1.84
23	Ociad1	OCIA domain-containing protein 1	0.03	0.83
24	Commd7	COMM domain-containing protein 7	0.04	1.26
25	Marcksl1	MARCKS-related protein	0.01	0.71
26	Sh3bp1	SH3 domain-binding protein 1	0.03	1.27
27	Spred1	Sprouty-related, EVH1 domain-containing protein 1	0.01	1.25
28	Slc7a5	Large neutral amino acids transporter small subunit 1	0.04	1.23
29	Tns3	Tensin-3 OS=Mus musculus	0.04	0.72
30	Celf1	CUGBP Elav-like family member 1	0.02	0.76
31	Arhgef1	Rho guanine nucleotide exchange factor 1	0.02	1.20
32	Gstt1	Glutathione S-transferase theta-1	0.02	0.59
33	Pigk	GPI-anchor transamidase	0.00	1.37
34	Dtnb	Dystrobrevin beta	0.00	1.40

35	Glmn	Glomulin	0.03	1.27
36	Vtn	Vitronectin	0.04	1.78
37	Tnfaip8	Tumor necrosis factor alpha-induced protein 8	0.02	0.80
38	Btf3	Transcription factor BTF3	0.01	0.71
39	Adcy5	Adenylate cyclase type 5	0.05	1.31
40	Pgrmc2	Membrane-associated progesterone receptor component 2	0.03	0.79
41	Igsf9b	Protein turtle homolog B	0.04	1.20
42	Plxna3	Plexin-A3	0.01	1.60
43	Rps28	40S ribosomal protein S28	0.03	1.51
44	Slirp	SRA stem-loop-interacting RNA-binding protein, mitochondrial	0.00	0.74
45	Tub	Tubby protein	0.05	1.24
46	Lrrc8c	Volume-regulated anion channel subunit LRRC8C	0.05	1.31
47	Sphk2	Sphingosine kinase 2	0.01	1.32
48	Abcg2	Broad substrate specificity ATP-binding cassette transporter ABCG2	0.04	1.42
49	Sytl2	Synaptotagmin-like protein 2	0.03	1.80
50	Ppp1r11	E3 ubiquitin-protein ligase PPP1R11	0.02	0.62
51	Ssbp1	Single-stranded DNA-binding protein, mitochondrial	0.04	0.80
52	Apip	Methylthioribulose-1-phosphate dehydratase	0.02	0.80
53	C1qb	Complement C1q subcomponent subunit B	0.01	1.43
54	Chmp1a	Charged multivesicular body protein 1a	0.05	0.76
55	Pon1	Serum paraoxonase/arylesterase 1	0.05	1.56
56	Eef1akmt1	EEF1A lysine methyltransferase 1	0.05	0.77
57	Igfals	Insulin-like growth factor-binding protein complex acid labile subunit	0.03	1.26
58	Bin2	Bridging integrator 2	0.04	1.52
59	Mrs2	Magnesium transporter MRS2 homolog, mitochondrial	0.01	1.45
60	Sec14l1	SEC14-like protein 1	0.05	1.20
61	Fads2	Acyl-CoA 6-desaturase	0.00	1.25
62	Dtx3	Probable E3 ubiquitin-protein ligase DTX3	0.01	1.28
63	Pitpnc1	Cytoplasmic phosphatidylinositol transfer protein 1	0.04	1.37
64	Ptgfrn	Prostaglandin F2 receptor negative regulator	0.04	1.35
65	Ttl	Tubulin--tyrosine ligase	0.04	1.31

66	Tmem115	Transmembrane protein 115	0.05	1.29
67	Dop1a	Protein dopey-1	0.00	1.29
68	Naa30	N-alpha-acetyltransferase 30	0.03	1.31
69	Smarcb1	SWI/SNF-related matrix-associated actin-dependent regulator of chromatin subfamily B member 1	0.04	0.81
70	Kcnj9	G protein-activated inward rectifier potassium channel 3	0.02	1.40
71	Tdrd3	Tudor domain-containing protein 3	0.04	1.20
72	Prrc2b	Protein PRRC2B	0.01	1.35
73	Txndc12	Thioredoxin domain-containing protein 12	0.02	0.74
74	Uckl1	Uridine-cytidine kinase-like 1	0.01	1.26
75	Slitrk1	SLIT and NTRK-like protein 1	0.05	1.47
76	Sdhaf2	Succinate dehydrogenase assembly factor 2, mitochondrial	0.02	1.38
77	Tyro3	Tyrosine-protein kinase receptor TYRO3	0.04	1.64
78	Ulk2	Serine/threonine-protein kinase ULK2	0.01	1.37
79	Kcnj11	ATP-sensitive inward rectifier potassium channel 11	0.00	1.45
80	Saraf	Store-operated calcium entry-associated regulatory factor	0.04	1.35
81	Ap4e1	AP-4 complex subunit epsilon-1	0.03	1.60
82	Sgpl1	Sphingosine-1-phosphate lyase 1	0.01	0.73
83	Tomm5	Mitochondrial import receptor subunit TOM5 homolog	0.00	0.82
84	Sephs2	Selenide, water dikinase 2 OS=Mus musculus	0.04	0.68
85	Gatb	Glutamyl-tRNA(Gln) amidotransferase subunit B, mitochondrial	0.01	1.26
86	Kctd3	BTB/POZ domain-containing protein KCTD3	0.01	1.23
87	Plpp1	Phospholipid phosphatase 1	0.01	1.98
88	Slc17a5	Sialin	0.01	1.25
89	Kank3	KN motif and ankyrin repeat domain-containing protein 3	0.04	1.38
90	Pet117	Protein PET117 homolog, mitochondrial	0.01	1.26
91	Efnb3	Ephrin-B3	0.05	1.39
92	Lpp	Lipoma-preferred partner homolog	0.05	0.67
93	Dlg5	Disks large homolog 5	0.02	1.21
94	Epm2a	Laforin	0.03	1.25
95	Mcoln1	Mucolipin-1	0.03	1.21
96	Slitrk6	SLIT and NTRK-like protein 6	0.04	1.32
97	Fbxo21	F-box only protein 21	0.05	1.39

98	Asphd1	Aspartate beta-hydroxylase domain-containing protein 1	0.01	1.62
99	Kin	DNA/RNA-binding protein KIN17	0.00	0.64
100	Mxra7	Matrix-remodeling-associated protein 7	0.02	1.86
101	Mpc1	Mitochondrial pyruvate carrier 1	0.02	0.70
102	Serf1	Small EDRK-rich factor 1	0.04	0.66
103	Wdr70	WD repeat-containing protein 70	0.00	0.73
104	Disp2	Protein dispatched homolog 2	0.02	1.37
105	Romo1	Reactive oxygen species modulator 1	0.03	0.74
106	Clnk	Cytokine-dependent hematopoietic cell linker	0.03	0.82
107	Ankrd63	Ankyrin repeat domain-containing protein 63	0.03	1.40
108	Ifit3	Interferon-induced protein with tetratricopeptide repeats 3	0.05	1.20
109	Asb6	Ankyrin repeat and SOCS box protein 6	0.03	1.45
110	Mfsd2a	Sodium-dependent lysophosphatidylcholine symporter 1	0.03	1.29
111	Tbc1d14	TBC1 domain family member 14	0.03	1.43
112	Csmd1	CUB and sushi domain-containing protein 1	0.03	1.27
113	Kcnab3	Voltage-gated potassium channel subunit beta-3	0.02	1.58
114	Cox17	Cytochrome c oxidase copper chaperone	0.02	0.68
115	Fut9	4-galactosyl-N-acetylglucosaminide 3-alpha-L-fucosyltransferase 9	0.02	1.35
116	Sel1l3	Protein sel-1 homolog 3	0.03	1.24
117	Flg2	Filaggrin-2	0.05	1.87
118	Coprs	Coordinator of PRMT5 and differentiation stimulator	0.01	1.24
119	Casp4	Caspase-4	0.04	1.42
120	Tbc1d22a	TBC1 domain family member 22A	0.01	2.31
121	Mgat4c	Alpha-1,3-mannosyl-glycoprotein 4-beta-N-acetylglucosaminyltransferase C	0.04	1.61
122	Rbm42	RNA-binding protein 42	0.04	0.77
123	Timm23	Mitochondrial import inner membrane translocase subunit Tim23	0.00	1.30
